# Post-COVID-19 Outcomes of Patients with Autosomal Dominant Polycystic Kidney Disease: A Multicenter Controlled Study

**DOI:** 10.3390/jcm15051850

**Published:** 2026-02-28

**Authors:** Serhat Karadag, Savas Ozturk, Nimet Aktas, Sinan Trabulus, Zeki Aydin, Hamad Dheir, Tolga Kuzu, Fatih Yilmaz, Yavuz Ayar, Irem Pembegul, Taner Basturk, Ruya Mutluay, Egemen Cebeci, Metin Ergul, Esra Akcali, Tuba Elif Ozler, Alper Azak, Mahmud Islam, Mustafa Arici, Kenan Ates

**Affiliations:** 1 Department of Nephrology, Haseki Training and Research Hospital, University of Health Sciences, 34265 Istanbul, Turkey; egemencebeci@hotmail.com; 2 Department of Internal Medicine, Division of Nephrology, Istanbul Faculty of Medicine, Istanbul University, 34093 Istanbul, Turkey; savasozturkdr@yahoo.com; 3 Department of Nephrology, Bursa Yüksek Ihtisas Training and Research Hospital, University of Health Sciences, 16310 Bursa, Turkey; nimetaktas@gmail.com; 4 Department of Internal Medicine, Division of Nephrology, Cerrahpasa Medical Faculty, Istanbul University—Cerrahpasa, 34098 Istanbul, Turkey; sinantrabulus@gmail.com; 5 Department of Nephrology, Darica Farabi Training and Research Hospital, University of Health Sciences, 41700 Kocaeli, Turkey; zekiaydindr@yahoo.com; 6 Department of Internal Medicine, Division of Nephrology, Sakarya Faculty of Medicine, Sakarya University, 54290 Sakarya, Turkey; hamaddheir@gmail.com; 7 Department of Internal Medicine, Division of Nephrology, Faculty of Medicine, Cukurova University, 01790 Adana, Turkey; tkuzu35@gmail.com; 8 Department of Nephrology, Antalya Atatürk State Hospital, 07040 Antalya, Turkey; fthylmz79@gmail.com; 9 Department of Nephrology, Bursa Faculty of Medicine, Bursa City Hospital, University of Health Sciences, 16110 Bursa, Turkey; yavuzayar@hotmail.com; 10 Department of Internal Medicine, Division of Nephrology, Faculty of Medicine, Malatya Turgut Ozal University, 44210 Malatya, Turkey; pembegulmd@yahoo.com; 11 Department of Nephrology, Sisli Hamidıye Etfal Training and Research Hospital, University of Health Sciences, 34371 Istanbul, Turkey; tanerbast@yahoo.com; 12 Department of Internal Medicine, Division of Nephrology, Faculty of Medicine, Eskisehir Osmangazi University, 26040 Eskisehir, Turkey; ruyamutluay@yahoo.com; 13 Department of Internal Medicine, Division of Nephrology, Faculty of Medicine, Kocaeli University, 41001 Kocaeli, Turkey; drmetinergul@gmail.com; 14 Department of Internal Medicine, Division of Nephrology, Faculty of Medicine, Mersin University, 33079 Mersin, Turkey; esratokman@yahoo.co.uk; 15 Department of Internal Medicine, Division of Nephrology, Faculty of Medicine, Amasya University, 05100 Amasya, Turkey; telifsenel@gmail.com; 16 Department of Nephrology, Balikesir Atatürk Education and Research Hospital, 10100 Balikesir, Turkey; dralperazak@gmail.com; 17 Department of Nephrology, Zonguldak Ataturk State Hospital, 67030 Zonguldak, Turkey; drisleem@gmail.com; 18 Department of Internal Medicine, Division of Nephrology, Faculty of Medicine, Hacettepe University, 06800 Ankara, Turkey; aricim@gmail.com; 19 Department of Internal Medicine, Division of Nephrology, Faculty of Medicine, Ankara University, 06230 Ankara, Turkey; kenan.ates@medicine.ankara.edu.tr

**Keywords:** COVID-19, chronic kidney disease, autosomal dominant polycystic kidney disease

## Abstract

**Background**: Patients with chronic kidney disease (CKD), including autosomal dominant polycystic kidney disease (ADPKD), have been reported to be at higher risk for adverse outcomes during the COVID-19 pandemic. We aimed to obtain the characteristics and outcome data obtained in the follow-up of patients with ADPKD who survived COVID-19 and to compare these data with a control group with ADPKD who were COVID-19 naive. **Methods**: In this national, multicenter observational study, adult ADPKD patients who survived COVID-19 were included, and ADPKD patients without a history of COVID-19 from the same outpatient clinics were selected as a control group. Baseline characteristics and post-COVID-19 first- and third-month data were recorded. **Results**: In this study, a total of 72 ADPKD patients from 16 centers were included in the study (COVID-19 group, *n*: 40, non-COVID-19 control group, *n*: 32). Fourteen (33.3%) patients in the COVID-19 group were hospitalized during active COVID-19. During the first and third months after COVID-19, none of the patients died in either group. Urinary tract infection was significantly higher in the non-COVID-19 group than in the COVID group in the third month (0% vs. 12.5%, *p* = 0.021). All other follow-up outcomes, including respiratory symptoms and initiation of kidney replacement therapy (KRT), were not different between the groups in the first and third months. The laboratory data of the groups in the first and third months were not significantly different. Hematuria and leukocyturia ratios were also not statistically significantly different between the groups. **Conclusions**: ADPKD patients who survive COVID-19 have no worse short-term outcomes than non-COVID-19 ADPKD patients, including respiratory symptoms, initiation of KRT, and death.

## 1. Introduction

Autosomal dominant polycystic kidney disease (ADPKD) is the most common genetic kidney disease, affecting approximately 1 in 1000 live births worldwide [1]. ADPKD forms cysts in the kidneys, leading to a range of complications, including hypertension, chronic kidney disease (CKD), and end-stage renal disease [2]. During the coronavirus disease 2019 (COVID-19) pandemic, patients with chronic diseases such as CKD were reported to have more adverse outcomes, including hospitalization, need for intensive care support, and death [3,4,5,6]. Few data are available in the literature on the importance of having ADPKD during the COVID-19 pandemic. Although some studies indicate that having ADPKD is an elevated risk of COVID-19 complications, there are also studies showing that ADPKD patients with COVID-19 do not result in different outcomes than non-COVID-19 ADPKD patients [7,8]. These studies include data during the active COVID-19 period. There are no published data that we can find on the outcome of ADPKD patients who have had COVID-19.

The pathogenesis of ADPKD is not directly related to immune disorders; thus, it may not be expected to cause more severe consequences in the post-COVID-19 period. However, outcomes may be worse in patients with advanced renal impairment. While prior studies have primarily examined the acute phase, the post-COVID recovery period may reveal persistent renal or systemic effects that remain under-characterized in ADPKD. Therefore, we aimed to evaluate the clinical characteristics and short-term outcomes of ADPKD patients who survived COVID-19 and to compare these findings with a COVID-19-naïve ADPKD control group.

## 2. Materials and Methods

This retrospective cohort study followed the Strengthening Reporting of Observational Studies in Epidemiology (STROBE) statement [9]. Ethical approval of the study was obtained from the Health Sciences University, Haseki Training and Research Hospital Ethics Committee (Date: 28 April 2021, protocol number: 12-2021).

### 2.1. Population and Setting

This study was designed as a retrospective controlled cohort study. The index date for the COVID-19 group was defined as the date of reverse transcriptase-polymerase chain reaction (RT-PCR) confirmation. For the control group, the index date was defined as the outpatient visit occurring in the same calendar month as the corresponding COVID-19 case to ensure temporal alignment of follow-up. COVID-19 cases were identified through the Turkish Society of Nephrology web-based registry, which prospectively collects national data on COVID-19 from voluntary nephrology centers. Patients included in the registry were known ADPKD patients followed in participating nephrology clinics who developed RT-PCR-confirmed SARS-CoV-2 infection and were subsequently reported by the treating nephrologist. COVID-19 infection was confirmed by a positive SARS-CoV-2 nasopharyngeal RT-PCR test. Patients diagnosed only by clinical or radiological findings without a confirmatory RT-PCR test were excluded from the COVID-19 group. Both symptomatic and asymptomatic RT-PCR-positive patients were eligible for inclusion. Data were collected from April 2021 to September 2021. A control group consisting of ADPKD patients without COVID-19 was formed from the same centers. COVID-19-free status was determined based on the absence of documented RT-PCR positivity and no recorded clinical diagnosis of COVID-19 in hospital electronic medical records at the time of selection. The first COVID-19-free ADPKD patient who came to the outpatient clinic of the participating centers after an ADPKD patient with COVID-19 was selected as the Control group, regardless of serum creatinine and glomerular filtration rate (GFR) values. To minimize selection bias, control patients were chosen from the same outpatient clinics and during the same time frame as the COVID-19 cases, ensuring comparable access to healthcare and follow-up frequency. Although individual matching was not performed, the two groups were similar in terms of age, sex, and major comorbidities, as shown in Table 1. All patients in both groups were followed with the same post-evaluation schedule (baseline, first month, and third month visits), using identical laboratory and clinical data collection procedures. The diagnosis, disease severity, and treatment of patients who had COVID-19 were managed with the guidelines recommended by the Ministry of Health [10].

The study excluded patients with end-stage renal disease, kidney transplant recipients, individuals under 18 years of age, and those with known kidney diseases other than ADPKD—such as diabetic nephropathy, hypertensive nephrosclerosis, glomerulonephritis, or NSAID-induced nephropathy. Additionally, within the COVID-19 group, patients without a confirmed positive nasopharyngeal SARS-CoV-2 reverse transcriptase-polymerase chain reaction (RT-PCR) test were also excluded. We also excluded patients with missing third-month outcome data after COVID-19. Potential control patients with documented COVID-19 infection during the study period or missing follow-up data were also excluded from analysis.

COVID-19 vaccination status was not consistently available in the registry database. During the study period (April–September 2021), vaccination in Turkey had begun with priority groups, including healthcare workers and older adults, but was not yet widespread across the general population; therefore, vaccination data could not be analyzed.

### 2.2. Measurements and Definitions

We gathered baseline characteristics, comorbidities and medications, blood pressure, height, weight, basic laboratory test results including glucose, urea, creatinine, albumin, C reactive protein (CRP), hemoglobin, proteinuria, urinalysis, and hemogram parameters from hospital records at the last outpatient clinic visit of the relevant center before the development of COVID-19. The same laboratory tests were recorded after the first and third months. All laboratory measurements were performed in the clinical laboratories of participating centers, each accredited by the Turkish Ministry of Health. Laboratory methods and reference ranges were consistent across centers. COVID-19-related outcomes were recorded from electronic hospital systems and verified by the treating nephrologist at each center to ensure data accuracy. Proteinuria was recorded both qualitatively, as measured by dipstick, and quantitatively by deriving from spot urine protein creatinine ratio or measured from 24-h collected urine protein amount. Hematuria was defined as the presence of 5 or more erythrocytes per high-power field. Leukocyturia was defined as 5 or more leukocytes per high-power field. In addition, information about the active period of COVID-19 [symptoms, chest computed tomography (CT), treatments for COVID-19, clinical severity of the disease according to the Ministry of Health guideline [10], inpatient or outpatient treatment, need for ICU, dialysis, and the duration of the hospitalization] was obtained. The data of patients included in the non-COVID group were obtained in the same month (including baseline visits) as the equivalent patient in the COVID-19 group.

For subgroup analysis, COVID-19-positive ADPKD patients were further divided into two categories according to their management during the acute infection: hospitalized and outpatient. A third group consisted of ADPKD patients without a history of COVID-19 (controls). All three subgroups were followed with the same post-COVID evaluation protocol, and laboratory parameters were obtained at baseline, first, and third months. These subgroup analyses according to hospitalization status were exploratory and conducted post hoc, as they were not prespecified in the original study design.

### 2.3. Follow-Up and Outcome

Primary endpoints were all-cause mortality, initiation of chronic kidney replacement therapy (KRT), and persistent respiratory symptoms at 1 and 3 months. Secondary endpoints included hospitalization during follow-up, urinary tract infection (UTI), lower respiratory tract infection, thromboembolic events, and laboratory parameters. We collected clinical data, including mortality, initiation of chronic Kidney replacement therapy (KRT), ongoing respiratory symptoms (cough and/or shortness of breath), hospitalization, need for home oxygen therapy, lower respiratory tract infection, and UTI development in our patient group at the 1st and 3rd months post-COVID-19. In addition, patients’ weight, blood pressure, creatinine, albumin, CRP, hemoglobin, proteinuria (qualitative and quantitative), hematuria and leukocyturia values in the urine sediment were obtained at 1 and 3 months. The same data were also obtained for the non-COVID-19 control group. In this control group, the data of each patient were obtained from the data in the same month as the patient with COVID-19 control. Ongoing respiratory symptoms were defined as the persistence of cough and/or dyspnea beyond 4 weeks after acute infection. UTI was recorded only in the presence of both compatible symptoms and either positive urine culture or physician-documented antibiotic prescription. All-cause mortality, new initiation of chronic KRT, and rehospitalization were recorded within the first and third months.

### 2.4. Statistical Analyses

In descriptive statistics, we presented numbers and percentages for categorical variables, and median and interquartile ranges (25–75%) for numerical variables. The normality of the variables was analyzed using Kolmogorov–Smirnov tests. The chi-square test was used to compare categorical variables in two or multiple groups. We used the independent t-test or the Mann–Whitney U test as appropriate to compare numerical variables. In multi-group comparisons of numerical variables, the analysis of variance (ANOVA) test was used for normally distributed numerical variables, and the Kruskal–Wallis test was used for numerical variables that were not normally distributed. The significance level was accepted as *p* < 0.05. Given the limited sample size, no multivariable adjustments were applied. Therefore, the analyses should be interpreted as exploratory and hypothesis-generating. Effect sizes (median differences and proportions) were examined to identify clinically meaningful trends even when statistical significance was not achieved. IBM SPSS Statistics for Windows, Version 26.0 (IBM Corp., Armonk, NY, USA) was used for statistical analysis.

## 3. Results

### 3.1. Demographic and Baseline Characteristics

A total of 74 patients’ data were obtained from 16 centers for the study. Two patients were excluded because third-month outcome data were missing. In the final, 72 patients (40 ADPKD patients who survived COVID-19, and 32 ADPKD patients without a COVID-19 control group) were included in the study. The main characteristics of both groups were almost similar (Table 1). Age, gender, ADPKD duration, extrarenal organ involvement, comorbidities, medications, and baseline laboratory tests were not different between the groups, except CRP [median: 6 (IQR: 2–36) mg/L) vs. 3 (IQR: 3–6) mg/L, *p* < 0.05] and hematuria [n (%): 14 (41.2) vs. 6 (18.8), *p* < 0.05], which were significantly higher in the COVID-19 group than COVID-19 free group.

### 3.2. The Acute Phase of COVID-19

The clinical severity of patients who had COVID-19 at the time of first admission to the hospital was as follows: asymptomatic disease 3 (7.5%), mild disease 27 (67.5%), moderate-severe disease 10 (25.0%). None of the patients had severe critical illness. Fourteen (33.3%) of them were treated as inpatients. The length of stay at the hospital was 10 (8–13) days. Acute kidney injury developed in 9 (64.2%) of the hospitalized patients (7 patients KDIGO stage 1, 2 patients stage 3). Nine (64.2%) patients were given glucocorticoids for COVID-19 in addition to symptomatic treatments. No patient was admitted to the intensive care unit.

### 3.3. Comparison of First and Third-Month Laboratory Values and Outcomes

There were no significant differences in laboratory parameters between the COVID-19 and non-COVID-19 groups at the first and third months (Table 2). During the first and third months after COVID-19, none of the patients died in either group. Only UTI was significantly higher in the non-COVID-19 group than in the COVID-19 group in the third month (0% vs. 12.5%, *p* = 0.021). All other follow-up outcomes, including initiation of chronic KRT, were not different between the groups in the first and third months.

### 3.4. Comparison of First and Third-Month Laboratory Values and Outcomes According to Hospitalization of COVID-19 Group

When the COVID-19 group was divided into two groups according to outpatient (26 patients) or inpatient (14 patients) treatment and compared with the COVID-19-free group (Supplementary Table S1); age and presence of recurrent urinary infection were higher in the inpatient group. All inpatient-treated patients had pneumonia on chest CT, and had a higher rate of dyspnea, cough, and moderate-severe illness than the outpatient-treated group at presentation. Baseline creatinine, CRP levels, rate of hematuria, and proteinuria were significantly higher in the inpatient group than in the other groups. When the outcomes of these groups were compared in the first and third months, there was no significant difference between these groups (Supplementary Table S2).

### 3.5. Additional Subgroup Analyses

**Figure 1:**
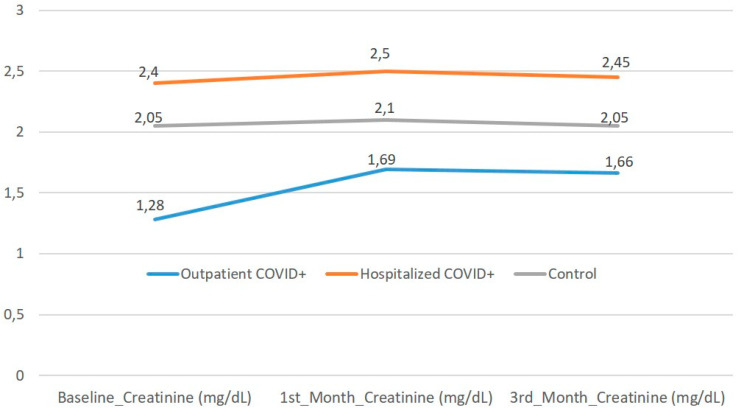
Longitudinal trend of serum creatinine levels in ADPKD patients with and without prior COVID-19 infection. Median serum creatinine levels (mg/dL) of three patient subgroups are shown across baseline, first-month, and third-month follow-up. Hospitalized COVID-19-positive ADPKD patients had higher baseline creatinine values compared to outpatients and non-COVID controls; however, no progressive renal deterioration was observed over the three-month post-COVID period. Lines represent group medians, and markers indicate time points (Baseline, 1st Month, 3rd Month).

**Figure 2:**
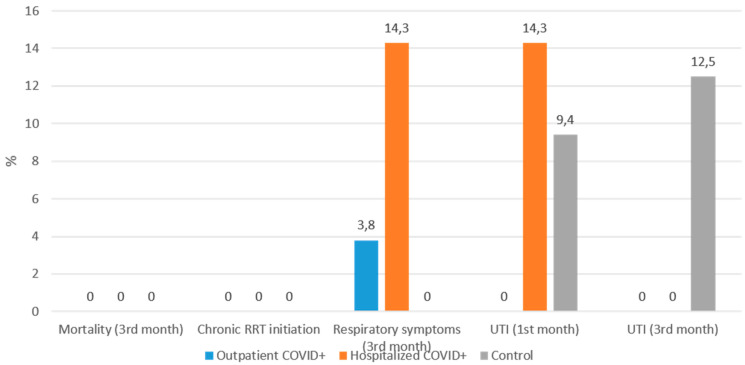
Comparison of short-term clinical outcomes among ADPKD subgroups. The proportions (%) of patients experiencing key post-COVID outcomes are displayed across three groups: Outpatient COVID-19-positive, Hospitalized COVID-19-positive, and COVID-19-naïve controls. No mortality or initiation of chronic kidney replacement therapy was observed in any group. The only statistically significant difference was a higher incidence of urinary tract infection (UTI) in the control group at the third month (*p* = 0.02).

Supplementary Table S1 summarizes baseline demographic and laboratory findings of outpatients, hospitalized COVID-19-positive ADPKD patients, and non-COVID controls. Hospitalized patients had significantly higher creatinine and CRP levels than both outpatients and controls (*p* < 0.05). Other parameters, including blood pressure, albumin, and proteinuria, were comparable among groups. Supplementary Table S2 shows short-term outcomes across the three subgroups. No mortality or chronic KRT initiation was observed during follow-up. UTI at the third month was more frequent in the control group (*p* = 0.02), while other outcomes showed no significant differences. Figure 1 demonstrates that serum creatinine levels remained stable from baseline to the third month among all three subgroups. Although hospitalized COVID-19-positive patients had slightly higher baseline creatinine levels, no progressive deterioration was observed during follow-up. The distribution of post-COVID events—including respiratory symptoms, UTIs, and need for hospitalization—was similar across all subgroups, with no mortality or chronic KRT observed in any group (Figure 2). A higher frequency of UTIs was noted in the control group at the third month (12.5%), which reached statistical significance (*p* = 0.02). These findings are consistent with the overall observation that ADPKD patients recovering from COVID-19 do not experience worsened short-term outcomes compared with COVID-naïve counterparts.

## 4. Discussion

The results of this study provided important insights into the outcomes of ADPKD patients who survived COVID-19 in the post-acute period. Our study found that mortality, initiation of chronic KRT, and respiratory symptoms were not significantly different in ADPKD patients COVID-19 survivors compared to non-COVID-19 ADPKD patients in the first three months of the post-COVID-19 period. Importantly, both groups were comparable in demographic and clinical characteristics at baseline, and follow-up evaluations were performed under identical conditions, which supports the internal validity of our findings. Moreover, subgroup analyses revealed that hospitalized COVID-19-positive ADPKD patients had higher baseline creatinine and CRP levels compared to outpatients and controls, reflecting greater inflammation and renal burden during acute disease. Nevertheless, post-COVID outcomes were similar among all groups, suggesting preserved renal function and a favorable short-term prognosis in ADPKD patients who recover from COVID-19. Although data specifically examining COVID-19 outcomes in ADPKD are limited, existing reviews suggest that while CKD disease in general increases the risk of adverse outcomes, patients with ADPKD do not appear to have substantially higher COVID-19-related mortality compared with CKD cohorts, supporting our findings of preserved short-term outcomes post-infection [8]. Given the reduced sample size within subgroups, these findings should be interpreted cautiously. The absence of significant differences during follow-up does not exclude potential clinically relevant effects, and the subgroup analyses should be considered exploratory and hypothesis-generating.

Recent longitudinal data by Lai et al. have provided additional insights into the long-term renal impact of COVID-19 among patients with ADPKD [11]. In that multicenter retrospective cohort of 103 patients, ADPKD individuals exhibited a more pronounced decline in renal function—reflected by increased serum creatinine and decreased eGFR—one year after COVID-19 infection compared to patients with CKD alone, particularly among those with baseline eGFR below 60 mL/min/1.73 m^2^. This observation suggests that while short-term outcomes appear similar between ADPKD and non-ADPKD CKD patients, long-term follow-up may reveal subtle but progressive kidney function deterioration in ADPKD following SARS-CoV-2 infection. Our findings, which demonstrate no significant renal worsening within the first three months, are therefore complementary to these long-term data and highlight the need for extended follow-up in this population.

While some numerical differences were observed in baseline characteristics and clinical outcomes, none reached statistical significance. This may be attributed to the limited sample size, which could have reduced the statistical power to detect modest but clinically meaningful differences.

Nevertheless, the overall absence of adverse post-COVID outcomes in the ADPKD group is consistent with previous reports suggesting that ADPKD does not inherently increase the risk of COVID-19-related complications. Our findings, therefore, support and extend the existing literature by providing new data from the post-acute recovery period. Cui et al. showed that in patients with ADPKD who were positive for COVID-19, the rates of hospitalization, ICU admission, ventilator requirement, and death were comparable with those of patients with other cystic kidney disease and cystic liver-only disease in a study including 61 veteran patients [7]. In a large cohort of patients hospitalized for COVID-19 previously published by our group, none of the 19 patients with ADPKD (4 kidney transplant patients, 2 predialysis CKD patients and 13 hemodialysis patients) died. However, these studies evaluated the outcomes during the active COVID-19 period, whereas our study evaluated the post-acute period outcomes [6]. A narrative review by Kutky [8] et al. found no evidence that patients with ADPKD are at higher risk for SARS-CoV-2 infection. Also, according to this study, people with renal dysfunction among those who develop COVID-19 have an increased risk of hospitalization, intensive care unit admission, and death, but ADPKD does not seem to specifically change the risk. However, none of these studies specifically evaluate the outcomes of ADPKD patients in the post-COVID-19 period. Consistent with broader COVID-19 literature, elevated CRP has been linked to adverse outcomes and organ dysfunction in infected patients. Smilowitz et al. demonstrated that higher CRP levels correlate with acute kidney injury, critical illness, and mortality among hospitalized COVID-19 patients, supporting the role of systemic inflammation in disease progression [12]. Furthermore, meta-analytic data indicate that higher CRP concentrations are significantly associated with greater disease severity and lower survival rates in COVID-19 [13]. These findings align with our observation that hospitalized ADPKD patients had higher CRP levels, though this did not translate into worse short-term renal outcomes in our cohort.

Our study also found that UTI was significantly higher in the non-COVID-19 group than in the COVID-19 group in the third month. No significant difference was observed in the first month. Although antibiotic exposure during acute COVID-19 could theoretically influence subsequent UTI incidence, antibiotic type and duration were not systematically recorded; therefore, this explanation remains speculative. Alternative explanations include differences in healthcare utilization patterns during the pandemic, variation in diagnostic intensity, or the possibility of a chance finding given the small number of events. Baseline prevalence of recurrent urinary infection and sex distribution were similar between groups; however, due to the limited sample size, these results should be interpreted cautiously and considered hypothesis-generating. Further studies are needed to confirm this finding and to evaluate the long-term effects of COVID-19 on UTIs in ADPKD patients. The lack of significant differences in laboratory data and the absence of statistically significant differences in the incidence of hematuria and leukocyturia ratios between the groups suggest that COVID-19 did not exacerbate ADPKD’s underlying pathophysiology.

This study has several limitations. First, the relatively small sample size and absence of major clinical events such as mortality or initiation of chronic kidney replacement therapy limit statistical power and increase the possibility of type II error. Therefore, modest but clinically meaningful differences between groups may not have been detected, and the findings should be interpreted as exploratory. Second, the follow-up period was limited to three months, which restricts the assessment of long-term renal progression in ADPKD. Although no significant short-term deterioration was observed, these results do not exclude the possibility of medium- or long-term renal consequences following SARS-CoV-2 infection, as suggested by recent longitudinal studies. Third, renal outcomes were primarily assessed using serum creatinine and proteinuria. Because ADPKD is a structural kidney disease, progression is more accurately reflected by longitudinal eGFR trajectories and imaging parameters such as total kidney volume (TKV), which were not systematically available in our dataset. Subtle structural progression or early decline in kidney function may therefore not have been fully captured. Fourth, despite temporal alignment and center-based control selection, residual selection bias cannot be entirely excluded. Differences in healthcare-seeking behavior during the pandemic and the possibility of unrecognized asymptomatic SARS-CoV-2 infection among controls may have influenced group comparability. Finally, vaccination status and prior SARS-CoV-2 infection history were not systematically recorded. During the study period (April–September 2021), COVID-19 vaccination in Turkey had primarily been administered to healthcare workers and high-risk groups and was not yet widespread in the general population. In addition, circulating SARS-CoV-2 variants evolved over time. These factors may limit the generalizability of our findings to later phases of the pandemic, characterized by higher vaccination coverage and different variant distributions. In conclusion, our findings suggest that ADPKD patients who survive COVID-19 do not appear to experience worse short-term (up to three months) post-acute outcomes compared with COVID-19-naïve ADPKD patients. Nevertheless, longer-term studies with larger cohorts are warranted to fully assess the long-term implications of COVID-19 in this vulnerable patient group.

## Figures and Tables

**Table 1:** Baseline characteristics, comorbidities, medications, and laboratory tests of both groups.

Parameter		COVID-19 Positive *n*: 40	COVID-19 Negative *n*: 32
Age (years), median (IQR)		45.5 (39–58)	46 (40–56)
ADPKD duration (Years), median (IQR)		10 (8–16)	10 (6–16)
Gender (Male), n (%)		19 (47.5)	17 (53.1)
Extrarenal involvement, n (%)			
Liver cysts		26 (65.0)	24 (75.0)
Intracranial aneurysm		1 (3.2)	2 (7.1)
Recurrent urinary infection		13 (33.3)	9 (28.1)
Heart valve involvement		3 (7.9)	0 (0)
Nephrolithiasis		11 (27.5)	7 (21.9)
Comorbidities, n (%)			
Diabetes mellitus		3 (7.5)	1 (3.1)
Hypertension		32 (80.0)	30 (93.8)
COPD		4 (10.0)	1 (3.1)
Ischemic heart disease		2 (5.1)	1 (3.1)
Heart failure		0 (0)	1 (3.1)
Cerebrovascular disease		1 (2.6)	1 (3.1)
Malignancy		1 (2.6)	0 (0)
Medications, n (%)			
ACE inhibitor		14 (35.0)	11 (34.4)
ARB		15 (37.5)	16 (50.0)
Calcium channel blocker		19 (47.5)	16 (50.0)
Beta-blocker		13 (32.5)	5 (15.6)
Other antihypertensives		4 (10.0)	7 (22.6)
Insulin		1 (2.5)	1 (3.2)
Oral antidiabetic		3 (7.5)	0 (0)
Statin		4 (10.0)	4 (12.5)
Antiaggregant		9 (22.5)	10 (31.3)
Anticoagulant		40 (100.0)	32 (100.0)
Tolvaptan		10 (25.0)	8 (25.0)
Cigarette, n (%)	Quit	14 (36.8)	13 (40.6)
	Smoker	1 (2.6)	2 (6.3)
	Non-smoker	23 (60.5)	17 (53.1)
Baseline (before COVID-19) data, median (IQR)			
Weight (kg), median (IQR)		78 (70–84)	75.65 (65–81)
Systolic BP (mmHg), median (IQR)		130 (120–140)	135 (125–142)
Diastolic BP (mmHg), median (IQR)		80 (80–87)	80 (76–90)
Glucose (mg/dL), median (IQR)		97 (84–109)	92 (86–98)
Urea (mg/dL), median (IQR)		49 (27–85)	54 (39–79)
Creatinine (mg/dL), median (IQR)		1.6 (1–2)	2.1 (1–3)
Sodium (mmol/L), median (IQR)		139 (137–141)	139.5 (138–141)
Potassium (mmol/L), median (IQR)		4.3 (4–5)	4.56 (4–5)
Calcium (mg/L), median (IQR)		9 (9–9)	9.17 (9–10)
Phosphorus (mg/L), median (IQR)		3.6 (3–4)	3.8 (3–5)
Parathormone (pg/mL), median (IQR)		112 (65–196)	115.6 (75–213)
ALT (U/L), median (IQR)		17 (13–21)	13.5 (11–20)
Albumin (g/dL), median (IQR)		4.2 (4–5)	4.2 (4–4)
* CRP (mg/L), median (IQR)		6 (2–36)	3 (3–6)
Hemoglobin (g/dL), median (IQR)		13 (12–14)	12.8 (12–15)
Leukocytes (/mm^3^), median (IQR)		6800 (5300–8680)	7100 (6350–8870)
Proteinuria (mg/day), median (IQR)		481 (125–866)	639 (132–961)
Proteinuria (dipstick negative), n (%)		21 (63.6)	17 (56.7)
* Hematuria, n (%)		14 (41.2)	6 (18.8)
Leukocyturia, n (%)		8 (23.5)	6 (18.8)

Abbreviations: IQR: Interquartile range, ADPKD: Autosomal dominant polycystic kidney disease, COPD: Chronic obstructive pulmonary disease, ACE: Angiotensin converting enzyme, ARB: Angiotensin II receptor blockers, BP: blood pressure, ALT: Alanine transaminase, CRP: C reactive protein. * *p* < 0.05.

**Table 2:** Comparative presentation of data regarding laboratory tests and outcomes in the first and third months after COVID-19.

Parameter	COVID-19 Positive *n*: 40	COVID-19 Negative *n*: 32
**First month after COVID-19**		
Weight (kg), median (IQR)	75.5 (68–83)	76 (71–85)
Creatinine (mg/dL), median (IQR)	1.69 (1–3)	2.1 (1–3)
Albumin (g/dL), median (IQR)	4.29 (4–5)	4.3 (4–5)
CRP (mg/L), median (IQR)	4 (2–14)	3 (2–4)
Hemoglobin (g/dL), median (IQR)	12.65 (11–14)	13.1 (12–15)
Proteinuria (mg/day), median (IQR)	400 (168–569)	380 (168–730)
Proteinuria (dipstick negative), n (%)	22 (57.9)	17 (58.6)
Hematuria, n (%)	13 (35.1)	5 (16.1)
Leukocyturia, n (%)	7 (18.4)	6 (19.4)
Death, n (%)	0 (0)	0 (0)
Need chronic KRT, n (%)	0 (0)	0 (0)
Respiratory symptoms, n (%)	3 (7.5)	0 (0)
Hospitalization, n (%)	0 (0)	1 (3.1)
Need of home oxygen therapy, n (%)	0 (0)	0 (0)
Lower respiratory tract infection, n (%)	0 (0)	0 (0)
Urinary tract infection, n (%)	2 (5.0)	3 (9.4)
Thromboembolic event, n (%)	0 (0)	0 (0)
**Third month after COVID-19**		
Weight (kg), median (IQR)	76.5 (70–82)	75.5 (66–82)
Creatinine (mg/dL), median (IQR)	1.66 (1–3)	2.05 (1–3)
Albumin (g/dL), median (IQR)	4.2 (4–5)	4.3 (4–5)
CRP (mg/L), median (IQR)	3.3 (2–7)	3 (1–4)
Hemoglobin (g/dL), median (IQR)	12.4 (11–14)	13.1 (12–14)
Proteinuria (mg/day), median (IQR)	422 (178–1600)	533 (240–975)
Proteinuria (dipstick negative), n (%)	23 (67.6)	14 (48.3)
Hematuria, n (%)	13 (36.1)	7 (21.9)
Leukocyturia, n (%)	8 (22.2)	9 (28.1)
Death, n (%)	0 (0)	0 (0)
Need chronic KRT, n (%)	0 (0)	0 (0)
Respiratory symptoms, n (%)	3 (7.5)	0 (0)
Hospitalization, n (%)	0 (0)	2 (6.3)
Need of home oxygen therapy, n (%)	0 (0)	0 (0)
Lower respiratory tract infection, n (%)	0 (0)	0 (0)
* Urinary tract infection, n (%)	0 (0)	4 (12.5)
Thromboembolic event, n (%)	0 (0)	0 (0)

Abbreviations: IQR: Interquartile range, CRP: C reactive protein, KRT: Kidney replacement therapy. * *p* < 0.05.

## Data Availability

The original contributions presented in this study are included in the article/Supplementary Material. Further inquiries can be directed to the corresponding author.

## Supplementary Materials

The following supporting information can be downloaded at: https://www.mdpi.com/article/10.3390/jcm15051850/s1, Table S1: Baseline demographic and laboratory parameters among outpatients, hospitalized COVID-19-positive ADPKD patients, and non-COVID controls; Table S2: Comparison of short-term outcomes among outpatients, hospitalized COVID-19-positive ADPKD patients, and non-COVID controls.
