# Supplementary material for: Post-COVID-19 Outcomes of Patients with Autosomal Dominant Polycystic Kidney Disease: A Multicenter Controlled Study
Source: J Clin Med. 2026 Feb 28;15(5):1850. doi: 10.3390/jcm15051850 (PMC12985428; doi:10.3390/jcm15051850)
Supplement: Supplementary file 1 [file jcm-15-01850-s001.zip › jcm-4144758-supplementary.pdf]

Supplementary Table S1.

Baseline demographic and laboratory parameters among outpatients, hospitalized COVID-19-positive ADPKD patients, and non-COVID controls

**Parameter**	**Outpatient COVID+ (n=26)**	**Hospitalized COVID+ (n=14)**	**Control (n=32)**	**p-value**
Age (years)	45 (38-56)	47 (41-58)	46 (40-56)	0.82
Male sex, n (%)	12 (46.1%)	7 (50.0%)	17 (53.1%)	0.88
Hypertension, n (%)	20 (76.9%)	12 (85.7%)	30 (93.8%)	0.21
Diabetes mellitus, n (%)	2 (7.7%)	1 (7.1%)	1 (3.1%)	0.73
Recurrent urinary infection, n (%)	8 (30.8%)	5 (35.7%)	9 (28.1%)	0.88
Creatinine (mg/dL)	1.28 (0.77-2.22)	2.40 (1.68-3.00)	2.05 (1.35-2.78)	0.04
Urea (mg/dL)	49 (27-85)	67 (42-96)	54 (39-79)	0.11
Albumin (g/dL)	4.39 (4.10-4.60)	4.10 (3.80-4.20)	4.30 (4.01-4.60)	0.72
CRP (mg/L)	3.00 (1.12-4.00)	8.00 (5.00-17.00)	3.00 (1.44-4.00)	**0.03**
Proteinuria (mg/day)	400 (168-569)	480 (190-730)	533 (240-975)	0.28
Hematuria, n (%)	9 (34.6%)	4 (28.6%)	7 (21.9%)	0.47
Leukocyturia, n (%)	5 (19.2%)	3 (21.4%)	9 (28.1%)	0.61
Weight (kg)	74.5 (64.5-82)	79.3 (73-81.5)	75.5 (65.5-81.5)	0.45
Systolic BP (mmHg)	130 (120-140)	135 (128-142)	135 (125-142)	0.33
Diastolic BP (mmHg)	80 (78-87)	82 (80-90)	80 (76-90)	0.41

Values are presented as median (25th-75th percentile) or n (%). p-values were calculated using Kruskal-Wallis test or chi-square test as appropriate. Significant results are in bold.

Supplementary Table S2.

Comparison of short-term outcomes among outpatients, hospitalized COVID-19-positive ADPKD patients, and non-COVID controls

Outcome	Outpatient COVID+ (n=26)	Hospitalized COVID+ (n=14)	Control (n=32)	p-value
Mortality (within 3 months)	0 (0%)	0 (0%)	0 (0%)	—
Initiation of chronic KRT	0 (0%)	0 (0%)	0 (0%)	—

Respiratory symptoms at 3rd month	1 (3.8%)	2 (14.3%)	0 (0%)	0.19
Hospitalization during follow-up	0 (0%)	1 (7.1%)	2 (6.3%)	0.32
Need for home oxygen therapy	0 (0%)	0 (0%)	0 (0%)	—
Lower respiratory tract infection	0 (0%)	0 (0%)	0 (0%)	—
Urinary tract infection (first month)	0 (0%)	2 (14.3%)	3 (9.4%)	0.18
Urinary tract infection (third month)	0 (0%)	0 (0%)	4 (12.5%)	**0.02**
Hematuria (third month)	9 (34.6%)	4 (28.6%)	7 (21.9%)	0.47
Leukocyturia (third month)	5 (19.2%)	3 (21.4%)	9 (28.1%)	0.61

Values are presented as n (%). p-values were calculated using chi-square or Fisher's exact test. Significant values are shown in bold.
